# Supplementary material for: Primary Care Professionals’ Self-Efficacy Surrounding Advance Care Planning and Its Link to Sociodemographics, Background and Perceptions: A Cross-Sectional Study
Source: Int J Environ Res Public Health. 2021 Aug 27;18(17):9034. doi: 10.3390/ijerph18179034 (PMC8430566; doi:10.3390/ijerph18179034)
Supplement: Supplementary file 1 [file ijerph-18-09034-s001.zip › ijerph-1336675-supplementary.pdf]

International Journal of
Environmental Research and Public Health

MDPI

Supplementary

Table S1. Supplemental table. Multivariate analysis. Logistic regression model (dependent variable ACP-SEs[1] >75 points).

	OR	IC95%	*p-value*
(Intercept)	0.06	0.03 – 0.11	**<0.001**
Have you participated in an ACP process with a patient? (Yes)	1.70	1.02 – 2.84	**0.043**
The ACP process contributes to improving the patients' quality of life (>8 points)	1.93	1.16 – 3.25	**0.013**
ACP facilitates knowledge of patients' values and preferences (>8 points)	2.24	1.12 – 4.76	**0.028**
Do you consider yourself to be sufficiently trained to carry out ACP processes? (≥ 8 points)	3.98	2.37 – 6.74	**<0.001**

[1] ACP-SEs= Advance Care Planning Self Efficacy Spanish; *p*-values < 0.05; Statistically significant differences have been marked in bold

Table S2. Supplemental table. Statistical probability of showing a condition and scoring the ACP -SEs [1] >75 points.

	Probability %
None condition	5.53%
Has participated in an ACP process	9.03%
ACP contributes to improving patients' quality of life (>8 points)	10.14%
ACP facilitates knowledge of patients' preferences and values (>8 points)	11.60%
Considers him/herself to be sufficiently trained to carry out ACP processes (>8 points)	18.89%
Having participated in an ACP process + ACP contributes to improving patients' quality of life >8 points + ACP facilitates knowledge of preferences and values >8 points + Considers him/herself to be sufficiently trained to carry out ACP processes >8 points	68.00%

[1] ACP-SEs= Advance Care Planning Self Efficacy Spanish.
